# Economic Rationality in Choosing between Short-Term Bad-Health Choices and Longer-Term Good-Health Choices

**DOI:** 10.3390/ijerph10115971

**Published:** 2013-11-08

**Authors:** David Campbell

**Affiliations:** Centre for Remote Health, Faculty of Health Sciences, School of Medicine, Flinders University, P.O. Box 4066, Alice Springs NT 0871, Australia; E-Mail: d.campbell@flinders.edu.au; Tel.: +61-889-514-744; Fax: +61-889-514-777

**Keywords:** smoking, alcohol abuse, health, behavioral choice, psychosocial determinants, human capital

## Abstract

Non-contagious, chronic disease has been identified as a global health risk. Poor lifestyle choices, such as smoking, alcohol, drug and solvent abuse, physical inactivity, and unhealthy diet have been identified as important factors affecting the increasing incidence of chronic disease. The following focuses on the circumstance affecting the lifestyle or behavioral choices of Aboriginal and Torres Strait Islander peoples in remote-/very remote Australia. Poor behavioral choices are the result of endogenous characteristics that are influenced by a range of stressful exogenous variables making up the psychosocial determinants including social disenfranchisement, cultural loss, insurmountable tasks, the loss of volitional control and resource constraints. It is shown that poor behavioral choices can be economically rational; especially under highly stressful conditions. Stressful circumstances erode individual capacity to commit to long-term positive health alternatives such as self-investment in education. Policies directed at removing the impediments and providing incentives to behaviors involving better health choices can lead to reductions in smoking and alcohol consumption and improved health outcomes. Multijurisdictional culturally acceptable policies directed at distal variables relating to the psychosocial determinants of health and personal mastery and control can be cost effective. While the content of this paper is focused on the conditions of colonized peoples, it has broader relevance.

## 1. Introduction

Non-contagious, chronic disease has been identified as a global health risk [1]. Poor lifestyle choices, such as smoking, alcohol, drug and solvent abuse, physical inactivity, and unhealthy diet have been identified as important factors affecting the increasing incidence of chronic disease [2]. Some have assessed poor behavioral choices as a result of people taking a short-term or myopic view of the individual’s own making, and that they should “reform themselves” [3]. Fang and Loury [3] suggest such commentary contains racial and class overtones and ignores the distal factors over which people have little or no control. Indeed, although poor lifestyle choices are the result of endogenous choice, individual endogenous status does not occur in isolation, but is impacted by exogenous factors.

Choices differ according to life experiences including the psychosocial determinants, culture and the community constraints in which they live. The following focuses on the circumstance and behaviours of Aboriginal and Torres Strait Islander people in remote-/very remote Australia; the term “remote” is used hereon. Aboriginal and Torres Strait Islander people suffer higher levels of chronic disease relative to the rest of the Australian population. Over the period 2005–2007 the life expectancy for Aboriginal and Torres Strait Islander males was 67.2 years (a gap of 11.5 years) and for Aboriginal and Torres Strait Islander females 72.9 years; resulting in a respective gap of 11.5 and 9.7 years, when compared with the rest of the Australian population [4]. The level of disadvantage for Aboriginal and Torres Strait Islander people in remote Australia is further demonstrated in school attendance rates, which are less than two thirds of the rest of the remote Australian population [5]. While attendance rates relate to individual choice, many of the schools attended by Aboriginal and Torres Strait Islanders are under-serviced [6]. While this material more closely relates to the conditions of colonised Indigenous peoples, broader based implications remain. That is, while specific differences exist between communities, commonalities exist across communities at higher levels of abstraction.

It is shown in this paper that poor health choices can be economically rational; especially when dealing with high levels of stress. Policies directed at lowering stress levels and altering the behavioral impediments and incentives people face can affect behaviour changes such as to reduce smoking and alcohol consumption and consequent improvements in health outcomes. The aim in this paper is to demonstrate the likely economic rationality of what are referred to as bad behavioral choices and how exogenous factors can influence an individual’s endogenous capacity in their choosing between alternative choices. Culturally acceptable policies directed at distal exogenous variables relating to the psychosocial determinants of health, involving improve personal mastery and control resulting in improved cost effectiveness, is discussed.

## 2. Method

The initial question regarding the economic rationality of bad health choices draws on personal knowledge from prior research regarding traditional economic circumstance of Aboriginal and Torres Strait Islander people [7,8,9] and multidisciplinary research in addressing chronic disease among Aboriginal people in remote Australia [10,11,12,13]. Economic rational choice was tested using an economic optimization model based on the tradeoff in choosing between (a) short-term lifestyle choices having negative health outcomes, or “short-term gratification”; and the alternative (b) long-term choice of “self-investment in human capital through education”—where education is an indicator of a range of positive longer-term behaviors (Appendix). The conclusion that bad health choices can be economically rational is supported through a multidisciplinary review of the literature on behavioral and health economics, primary health care, psychology and education. A working paper was forwarded to academics in the above disciplines, who are involved with Aboriginal and Torres Strait Islander health, for critiquing prior to preparation and submission of the final paper.

## 3. Economic Behavioral Choice

### 3.1. Self-Investment

Behavioural choice is assumed to be discrete and consistent, with continuous trade-offs between choices. This assumption is defendable across populations [14].

Self-investment in human capital through education can be considered according to the initial cost of capital investment, the expected annual rate of return on capital investment, the duration of capital investment, and investor time preference. These factors are considered in relation to behavioural choice. A positive correlation exists between improved health outcomes and education [1,15,16,17,18]. Improved health outcomes as a result of changes in lifestyle choice are likely to improve the quality and duration of human capital investment.

Extending Becker’s [19,20] work, Grossman [21] viewed education as an investment of market inputs and time in a human capital stock that produces an output of “healthy time”. Education improves income-earning potential and the capacity of individuals to make better life choices and improved efficiency in the use of resources [15]. The present value of capital investment is increased by minimising up-front opportunity costs and extending the time span of the flow of benefits that can be obtained by investing early in life; and when the opportunity cost of time spent in the class-room is less than it would be later in life.

Time enters and affects choice as a cost in achieving an expected healthier life, as occurs with social interactions, exercise, and education. Those expecting a longer lifespan are more likely to self-invest in education [15,22,23]. Expected life span is influenced by the observed life span of relations and community members [16]. Life span is affected by a range of factors in addition to genetic disposition [13]; including pre- and postnatal condition [24]. For example, improved survival of infants following the United Nations Expanded Programme on Immunization in Sub- Saharan Africa (Malawi, Tanzania, Zambia and Zimbabwe) influenced expectant mothers to improve their own nutrition for pre- and postnatal children [25]. 

Individual demand for education is derived from an expected improvement in income, status, health outcomes and a range of other market and non-market derived benefits. Non-market benefits of education and increased knowledge can lead to better behavioral choice, such as reduced smoking [21,23] and improved efficiency in the use of market and non-market sourced resources [26].

### 3.2. Assessing the Future: Time Discounting, Risk/Uncertainty, Volitional Control and Stress

**Figure 1:**
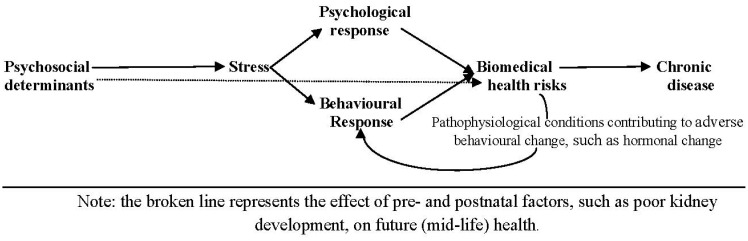
The pathway from the psychosocial determinants of health to chronic disease.

In choosing between good health choices or bad health choices there are immediate and longer-term opportunity costs. In choosing good health choices such as education, the benefits of improved income and health occur in the future, with a resulting delay in utility, while the costs are immediate. With bad health choices, or short-term gratification, the initial costs and benefits enter directly into the utility function. In this instance the negative health impact on the utility function occurs in the future. In sum, the positive impacts of good health choices are delayed, with the costs up front; while the negative health impacts of bad health choices are delayed with the benefits up-front. Depending on how the future is assessed, it can be rational for a consumer to make choices that have negative long-term health effects. That is, it is possible for economically rational utility-maximizing Aboriginal and Torres Strait Islander people to make decisions leading to a negative feedback loop and an increase in the expected life gap—as is later shown and discussed in relation to Figure 1.

The assumption of neutral interchangeability between time periods does not hold when the comparison involves making choices involving uncertainty, rather than certainty. In-which-case, a disproportionate discount is given to uncertainty [27,28,29,30,31]. The decisions made by a social epidemiologist, government administrator or politician, involve large populations for which probable outcomes and probable pay-offs are known and involve risk rather than uncertainty. That is, social epidemiologists are operating in a world in which the “experiment” is repeatable, and the value of the expected outcome can be estimated using compound probabilities—although the professional reputation of the decision maker might be affected if they were to make the wrong choice. Individual lifestyle decision makers are dealing with uncertainty, with possible financial or physical constraints limiting their capacity to repeat the “experiment”. Aboriginal and Torres Strait Islander people are more likely to be under greater resource limitations and budgetary constraints and live in a more costly environment than most Australians. As a result, they are less able to “repeat the experiment”, and consequently deal with higher levels of uncertainty than most Australians.

#### Assertion of Self-Control

An important capacity necessary to self-invest in human capital is the individual’s capacity to self-regulate, or to apply volitional control and willpower. Muraven and Baumeister [32] described self-control as an extension of control over the self by the self to maximize long-term best interests. They described capacity to apply self-control as being used and consumed any time an individual self-initiates, alters, or stifles a response. That is, those who are undergoing stress have less capacity to delay self-gratification; while the erosion of willpower due to stress is accumulative. Ozdenoren *et al*. [33] commented that there is evidence to suggest “that the exercise of self-control draws on a limited and fungible cognitive resource—a resource that is often called willpower”.

Cox [34] discussed how stress may be associated with behavioral outcomes of two types: direct responses, sometimes termed “self-medication” [35], such as smoking and excess alcohol; and secondary responses due to hormonal variation, such as higher cortisol levels, with lower capacity for relatively lower income groups to be persistent in carrying through tasks and to self-regulate behavior [36]. In such circumstance, smoking and alcohol consumption are used as a form of self-medication for stress; even if smokers are fully aware of longer-term negative impacts [37].

The high level of stress suffered by Aboriginal and Torres Strait Islander people in remote Australia [38] leads to a higher discount rate [39]. Indeed, the decision process might be likened to the economic minimax strategy when behavior is consistent with optimizing behavior under the assumption of the worst outcome.

## 4. Exogenous Variables Affecting Stress and Poor Educational Outcomes

### 4.1. Extending the Utility Maximization Model

The endogenous variables affecting behavioral choice can be affected by a range of exogenous variables and behavioral impediments. These can range from budgetary constraints, the quality of teaching including cultural compatibility, home and living environment, pre-school preparation, pre and post-natal nutrition, social interactions, through to the level of control. Such factors, as discussed in the following, can have a positive or negative influence on endogenous characteristics and ultimately behavioral outcomes.

### 4.2. Influence of the Psychosocial Determinants

#### 4.2.1. Culture

Culture is an important aspect of self-identification [40]. The higher levels of suicide of Aboriginal and Torres Strait Islanders is associated with cultural loss [41]. Schooling can result in stressors between traditional cultural patterns and the cultural patterns of western schooling [38], resulting in a decrease in Aboriginal and Torres Strait Islander school attendance [42,43]. Nasir and Hand [44] noted that educational frameworks based on social and cultural processes are central to learning. Dockery [45,46] examined the statistical association between wellbeing and educational attainment for Aboriginal and Torres Strait Islanders. He observed a positive relationship between cultural strength and improved wellbeing, educational attainment and workforce participation.

Positive self-identity has been found to be an important factor affecting Aboriginal and Torres Strait Islander classroom engagement; when this involves the student in constant reassessment according to their own cultural norms [39]. Similar results were observed for Canadian Native Americans [47,48]. While in New Zealand the introduction of the Māori-based Māori Educational Strategy 2008–2012 [49] provides an example of the benefits of integrating school experiences with the students’ cultural norms. Māori immersion curriculums have grown, where Māori students can attend Māori immersion schools and bilingual units and classes. The same model is being effectively applied to the delivery of health services to Māori people [50].

#### 4.2.2. Stress

Stress is a component along the pathway from the psychosocial determinants of health to chronic disease. The Macquarie Dictionary [51] defines stress as: “a disturbing physiological or psychological influence which produces a state of severe tension”. Stress has been likened to an “inverted U” [52]; where some stress (strain or challenge) is necessary for optimal performance. Others suggest stress is different to that affecting motivation and states of arousal, workfullness, alertness and vigor [53].

In discussing the impact of the psychosocial determinants of health, Krieger [54] describes how psychosocial factors can influence the multiple sectors of human wellbeing and stress in affecting “both behavioral and endogenous biological responses to human interactions”. In this, psychological stress is identified as a result of “despairing circumstances, insurmountable tasks, or lack of social support”. These stressors, she suggests: “(a) alter host susceptibility or become directly pathogenic by affecting neuroendocrine function; and/or (b) induce health damaging behavior (especially in relation to use of psychoactive substances such as smoking, alcohol, drug and solvent abuse, diet, and sexual behaviors)”. She further explains the role of social cohesion as either a positive or a negative factor supportive of stressful circumstance and appropriate or inappropriate behavior (see Figure 1).

Figure 1 shows the complex pathway, described by Krieger [54], from social disadvantage to stress to biomedical health risks and chronic disease, via a dual pathway involving psychological responses and behavioral responses to stress and poor behavioral choice. Stress is likely to occur at higher levels of adversary, such as in circumstances involving perceived loss of control [55]. Statistics Canada [56], for example, showed an inverse association between high personal stress levels with health. While work stress was linked to negative life events, chronic strain, lack of closeness and a loss of sense of mastery (or control). Important to the argument in this paper are the psychosocial factors contributing to poor behavioral choices and pathophysiological conditions. Such responses are a result of the influence of hormonal reactions to the response of the hypothalamus to exogenous stimuli. Flinn and England [57], for example, observed that “[c]hronic stress and high average cortisol levels are associated with frequency of illness, a stress–health relation suggested by temporal associations”.

These results are consistent with the Whitehall I and Whitehall II studies [58]. The Whitehall studies were designed to explain the decrease in expected life span among British public servants with decreasing status within the public service. In the Whitehall I study, less than 25% of the difference in health outcomes with decreasing workplace status was explained by poor behavioral choices. In the Whitehall II study more than 50% of the difference in health outcomes was explained by decreasing individual opportunity to exert control in the workplace—emphasizing the role of the “psychological response”, as shown in Figure 1. The follow-up Whitehall II studies show poor workplace decision authority, high job demand, effort-reward imbalance and associated work-based stress increasing with lower work status. Stansfeld *et al*. [59] and Schnall *et al*. [60] observed increased risk of psychiatric disorders on this basis. The same results are observed outside of the workplace [61,62,63].

#### 4.2.3. Aboriginal and Torres Strait Islander Experience

Continued loss of control along with a history of psychosocial factors, have ongoing effects on individual and community stress levels. Aboriginal and Torres Strait Islander people suffer higher levels of stress than the rest of the Australian population. In 2008, 65% of Aboriginal and Torres Strait Islander children (4–14 years) reported at least one stressful event for the previous 12 months—twice that reported by other Australians [2].

The effect of the loss of self-control has been reviewed among Canada’s Native people [64,65] and the Elcho Island Aboriginal community off northern Australia [66]. All three studies showed decreasing health with decreasing control. Tsey *et al*. [67] discuss the results of the Whitehall studies and the consistency of these studies with the lack of control experienced by Aboriginal people. Daniel *et al*. [66] observed the high levels of suicide among young Aboriginal males in north-eastern Arnhem Land as an indicator of the lack of emotional and psychological wellbeing and low levels of mastery and control.

The relative importance of control is emphasized by the experience of the Utopia Aboriginal community in central Australia, for whom the socioeconomic indicators are lower than that generally observed for other Northern Territory Aboriginal communities. Yet, their health outcomes more closely approximate the Northern Territory non-Aboriginal community than the Aboriginal community [68]. The better health outcomes were attributed to the greater level of control community members were able to assert over their lives.

Garvey [69] identified a history of denial of humanity, existence and identity for Aboriginal and Torres Strait Islanders as a source of intergenerational stress. That many of these risk factors “… lie outside the ambits of mental health services and require long-term, sustained efforts across multiple sectors of the community, emphasizes the need for collaborative interjurisdictional partnerships”. Paradies [70,71], in research carried out in Australia regarding Aboriginal and Torres Strait Islanders and for minorities in the United States of America, observed the negative impact of racism on health.

An important factor affecting control is the greater impact for Aboriginal and Torres Strait Islander people of government policies and institutions than exists for the remainder of the community [72]. The Australian Government’s Northern Territory Emergency Response (“the Intervention”) in 2007 [6] is a contemporary example of this. The intervention, which remains in place at the time of writing, applies across Aboriginal communities in the Northern Territory regardless of the steps undertaken by the communities. The denial of Aboriginal and Torres Strait Islander people’s goals and loss of community control as a result of the intervention is likely to result in further trauma.

Stress has been observed as a barrier to quitting smoking among Aboriginal and Torres Strait Islander people in western Sydney [73]. For Aboriginal Health Workers in remote Australia, whose role includes advising on the health risks of smoking, stress is a barrier to their quitting. Stress factors included nicotine addiction, grief and loss, social connections including domestic disputes, work based stressors, and racism [37]. 

Although cultural differences can influence differences between peoples in their response to increased income, increased income has been found to have the greatest marginal impact on wellbeing for low income earners [74]. While mental health is positively related to socioeconomic status [75], recovery from a disability, and consequent stressors, was observed as being slower for lower income earners.

### 4.3. Factors Affecting School Attendance and Classroom Attention

#### 4.3.1. Early Life Factors

Early life factors are linked through the parents’ psychosocial environment. These factors affect the child’s cognitive abilities, longer-term or secondary behavioral choice and immediate and longer-term health [36]. The capacity of children to engage in and benefit from school attendance is influenced by maternal nutritional status preceding and at the time of conception, by the pre- and postnatal environment, and early childhood environment [76]. These influences can include pre- and postnatal responses to poor diet, and whether the mother suffers from a cardiovascular disease condition during pregnancy [77,78,79,80]. Recent research indicates an early life gene-environment interaction contributing to later life health risks, where low and excessive birth weights have negative influence on later life outcomes [81,82].

These early influences are represented by the broken line in Figure 1, where the secondary feedback is represented by the line from biomedical health risks to poor behavioral responses. The environmental factors impinging on a child’s socio-emotional development include diet, crowding, noise, and substandard housing conditions. Children from low-income families, exposed to these conditions, have higher systolic and diastolic blood pressure, and higher cortisol, epinephrine and norepinehrine readings; indicating higher stress levels than their middle class cousins [36].

Such differences indicate decreased capacity for lower socioeconomic groups to carry through tasks due to poor self-regulation, resulting in poor attention to immediate health factors such as smoking, alcohol, drug and solvent abuse, physical inactivity, unhealthy diet and to longer-term factors such as education. Dunbar and Scrimgeor [83], for example, noted the negative effect of poor hearing on the uptake of classroom lessons by Aboriginal children in remote Australia. As an impediment to classroom attention, health impediments such as poor hearing, is a further burden on individual capacity to self-regulate.

Flinn and England [57], in a longitudinal study in the Commonwealth of Dominica, noted the interconnection between childhood stress and caretaker support by monitoring changes in cortisol levels. They found poor relationships (including family conflict and residential change) between the child and their caretaker, along with peer group and work pressures, high disease and low nutrition levels, led to increased cortisol levels. The authors found increased cortisol levels to be associated with immune suppression, inhibited growth, psychological problems and energy depletion.

#### 4.3.2. Pre-Preparation for Schooling

Heckman and Masterov [84] highlighted the importance of developing cognitive skills at a very early age, as gaps in cognitive ability remain constant after age eight. They concluded that policies to supplement child-rearing resources for disadvantaged families will not only reduce inequality, but will result in economic pay-offs through improved health, educational attainment and decreased antisocial behavior. 

Increasing emphasis is given in Australia to the role of the home environment in preparing children for school. Zubrick *et al*. [85] recommend the engagement of Aboriginal parents and caregivers as educators of their children in the first five years of life. Docket *et al*. [86] emphasised the important role of school readiness for Australian Aboriginal and Torres Strait Islander children. Important to this is an understanding by guardians of what is required in preparing children for “school readiness”.

#### 4.3.3. Affecting Effort in the Classroom

Akerlof and Kronton [87] noted that increased investment in education did-not always result in improved outcomes as students may place a higher value on their peer group relationship than their own academic performance. Students, they suggest, think of themselves and of others according to different social categories and that individual students gain utility when their own actions and the actions of others enhance their own self-image. Important to self-esteem is the congruence between the students’ social category/self-image and the social/school environment. Such congruence is influenced by the students’ ascriptive characteristics, including social background and capabilities. Students maximize their utility by implicitly choosing their social category and then choosing the educational effort according to the norms of their chosen category. Accordingly, the authors conclude, that the level of student engagement in education (time and effort) is influenced by the degree of social difference in their school environment rather than their expected future wage.

Malin [88] noted a two-way relationship between health and education for Aboriginal children, with a failure in health outcomes and school attendance due to lack of control within and outside of the classroom. She suggested that the subjective experience of discrimination, by provoking particular responses such as anger, cynicism and anxiety, may generate stresses. Although the causative relationship proposed in this paper is that the line of causality proceeds from stresses to stress to behavioral responses, this still leads, as Marlin suggests, to cardiovascular reactivity, high blood pressure and negative health outcomes. Berkman and Kawachi [89] found the most successful interventions for minimizing risk factors are those that incorporate social and organizational interaction and support.

The acceptance of poor academic performance can have a cumulative effect leading to limited access to lifelong learning, and lost employment and economic opportunities [85]. The quality of teaching for Aboriginal and Torres Strait Islander people in remote Australia has in many instances been below that received by other Australian children. This, in addition to the class-room environment failing to engage with the experience and culture of Aboriginal and Torres Strait Islander students [90]. The often harsh schooling experience of parents and older family members means carers may not be supportive of school attendance, and fail to engage children in preschool preparation. While the observed poor work opportunities of community elders, means children may question the benefits of schooling [90]. Zubrick *et al*. [85] suggest that educators should collaborate with parents and caregivers and encourage their participation in ensuring educational standards and performance and the needs of students are met.

## 5. Discussion and Conclusion

High stress levels have been shown to affect the selection of short term poor lifestyle choices in preference to the selection of longer term benefits through self-investing in education. It is helpful to identify the structural and causal interrelationships if we are to access their effect on stress. According to Anjzen’s [91] Theory of Planned Behaviour (TPB), intended and ultimate behavior can be explained according to: (a) structural norms, involving the influence of an individual’s attitude to a behavior and their capacity to handle stress, as per the endogenous factors discussed in Section 2; (b) the subjective norms, involving the influence of others on an individual’s behavioral intention; and (c) the effect of perceived behavioral control on initial intention and ultimate behavior; when (b) and (c) are exogenous influences affecting individual intention and ultimate behavior. Stress, then, is an important endogenous condition affecting intention and behavioral choice; when stress levels are the result of exogenous factors, as explained by subjective norms and perceived behavioral control.

According to the TPB, perceived volitional control is a necessary condition for intention and behavioral outcomes. Perceived behavioral control is a function of past experiences and existing conditions including feedback mechanism of the hormonal response to stress [55,91]. Stress levels are the result of psychosocial factors, control and the possible mitigation of stress through social support and social interactions. Such factors must be considered within the context of the community, especially for isolated Aboriginal and Torres Strait Islander communities in remote Australia. In which case, personal resilience to the psychosocial determinants of health and community resilience are likely to be closely interrelated. Closely related to the development of community resilience is the development of social capital, reciprocity, collective action, interpersonal interaction and collective efficacy [47,48].

In-addition-to-which, outcomes are a function of achievable as well as perceived control, and can be an impediment to behavioral outcomes [55]. Cox [34] discussed how stress may be associated with behavioral outcomes of two types: direct responses, sometimes termed “self-medication” [35], such as smoking and excess alcohol; and secondary responses due to hormonal variation, such as higher cortisol levels, with lower capacity for relatively lower income groups to self-regulate behavior and to be persistent in carrying through tasks [36]. As shown in Figure 1, hormonal variation is likely to result in a feedback affecting lifestyle behavioral choice.

It appears that health targeted expenditures are primarily focused on meeting proximate based risk factors and disease conditions, when the expected primary global cause of poor health are preventable. It is shown that distal factors and ongoing control are important to improving expected life outcomes through decreased stress and likely behavioral change. This result is consistent with Dockery’s [45,46] results in that Aboriginal and Torres Strait Islander people possessing cultural strength are more likely to engage in education and work. As shown, ongoing behavioral control is likely to result in a reduction in bad behavioral choices including reduced smoking and alcohol consumption.

A possible economic advantage in addressing the distal psychosocial factors is the possibility of cost efficiencies through scoping economies involving multiple benefits [10,12]. Research carried out in joint participation with an Aboriginal community of nearly 1,300 people in tropical Northern Territory showed decreased incidence of diabetes, renal disease and hypertension with increasing participation in traditional caring for country [92,93,94]. These results were estimated to have brought about annual savings in primary health care of $280,000 [11]. These savings were in addition to a range of public good benefits such as the biosequestration of greenhouse gases, maintenance of biodiversity and the mitigation of smoke and dust storms, which have physiological consequences and are a vector of disease [10,13]. Such activities also reinforce cultural connection, self-worth, and the capacity to self-regulate. 

These results show that the cost effectiveness of policies directed at changing the relative advantage of long-term benefits over short term benefits can be achieved by addressing distal psychosocial causative agencies. We can therefore expect greater productivity over a longer life span, and delays in medical costs. In-addition-to-which are the possibilities of scoping economies. Such scoping economies would occur through the multiple benefits achievable through educational attainments, plus more specific benefits, such as in the above example. Although not discussed here, is the likely-hood of complementary economies. The possibilities of achieving such economies, in the context of remote Australia, are discussed in Campbell [10] and Campbell *et al*. [12].

It has been shown that short-term bad health choices in preference to long-term good health choices can be economically rational. The incidence of bad health choices is likely to increase with increasing levels of stress and loss of volitional control. It is shown that policy interventions directed at exogenous variables such as the distal psychosocial determinants of health can assist in changing the variables affecting endogenous choice. It is worth observing that in general terms the approach proposed here, in regard to the prevention and mitigation of smoking, excessive alcohol and solvent abuse includes a whole of life approach in addition to specific targeting.

## Appendix: Testing Economic Rational Choice in Choosing between Poor Behavioral Choice and Education

Carson *et al*. [1] ([38] in the main article) observed the health of Aboriginal people as a function of exogenous and endogenous factors. These two sets of factors are represented by **X**_j,k_: “j” indicates events and conditions exogenous to the individual occurring independent of individual choice; “k” represents self-initiated, or endogenous behavioral choice influenced by exogenous variables.

Changes in the initial expected long-term disposition of Aboriginal and Torres Strait Islander health (H) as a function of exogenous events and endogenous behavioral choice, is:

ΔH = f(**X**_j,k_)
(1)

Exogenous conditions and endogenous choice are assumed to have either positive health outcomes, when j, or k = 1, or negative health outcomes when j, or k = 2. Initially, it is assumed that exogenous conditions are neutral with j held constant, with expected change in life span a function of behavioral choice.

Endogenous choice is classified according to whether there is a negative or positive effect on Aboriginal and Torres Strait Islander health and expected life span. That is ∂H/∂**X**_.,1_ > 0 and ∂H/∂**X**_.,2_ < 0; assuming: **X**_.,1_ ≥ 0; **X**_.,2_ ≥ 0.

Choices are made on the basis of individual preference so as to maximize utility (U) on the basis of choosing between education, **X**_.,1_, or bad behavioral choices, **X**_.,2_ within a budgetary constraint (B), where C_k_ is a vector of costs:

Max. U = U(**X**_.,1_, **X**_.,2_)
Subject to: f(C_k_, **X**_.,k_) ≤ B

A utility-maximizing decision maker will allocate their budget in choosing between **X**_.,1_ and **X**_.,2_, based activities such that the marginal benefit equals the marginal opportunity cost of the foregone alternative choices: ∂U/∂**X**_.,1_ = ∂U/∂**X**_.,2_.

The positive impacts of **X**_.,1_ are delayed, with the costs up front; while the negative health impacts of **X**_.,2_ are delayed with the benefits up-front. Depending on how the future is assessed, it can be rational for a consumer to make choices that have negative long-term health effects. That is, it is possible for economically rational utility-maximizing Aboriginal and Torres Strait Islander people to make decisions in favor of bad behavioral choices that lead to a negative feedback loop and an increase in the expected life gap. Or, ∂U/∂**X**_.,2_ > 0, even when ∂H/∂**X**_.,2_ < 0.
